# Detection of heat shock protein 70 in choroidal neovascular membranes secondary to age related macular degeneration

**DOI:** 10.1186/1756-0500-4-115

**Published:** 2011-04-08

**Authors:** Andreas PW Jöres, Dörthe Carstesen, Gabriele Thumann, Peter Walter, Andreas WA Weinberger

**Affiliations:** 1 RWTH Aachen University, Department of Ophthalmology, Pauwelsstr. 30, 52074 Aachen, Germany; 2 Augenklinik Starnberg, Josef-Jägerhuber-Str.7, 82319 Starnberg, Germany

## Abstract

**Background:**

Heat shock proteins are acute phase proteins that are upregulated in inflammation or following thermal stress. We analyzed the presence of the heat shock protein 70 (Hsp 70) in choroidal neovascular (CNV) membranes secondary to AMD after treatment with verteporphin photodynamic therapy (PDT) or transpupillary thermo therapy (TTT) to determine whether treatment correlated with the presence of Hsp70.

**Results:**

CNV membranes were removed by pars plana vitrectomy (ppV) and subretinal extraction. The membranes were analysed by light microscopy and the presence of Hsp 70 was examined using histochemistry. HeLa Cells served as controls.

Of the 14 membranes analysed 11 were Hsp70 positive and 3 negative. In the no pre-treatment group of 8 membranes 6 were Hsp70 positive and 2 negative; in the PTD group all 4 membranes were positive and in the TTT group 1 membrane was positive and 1 membrane was negative for Hsp70.

**Conclusion:**

Hsp70 is present in the most CNV membranes secondary to AMD. Pre-treatment of the membrane with PTD or TTT does not appear to influence the expression of Hsp70.

## Background

Choroidal neovascularization (CNV) is the leading cause of severe visual impairment in patients with age-related macular degeneration (AMD) since when left untreated CNV leads to disciform scarring of the macula [1,2].

Several therapeutic strategies have been attempted to reduce the destructive effects of CNV membranes and stabilize vision. Since 2006 the preferred treatment for neovascular AMD is the intravitreal injections of inhibitors of VGF's especially the monoclonal antibodies bevacizumab (Avastin^®^) and ranibizumab (Lucentis^®^) [3,4]. Photodynamic therapy (PDT), submacular surgery and laser procedures have become second line options. While argon laser photocoagulation destroys the CNV and overlying retina, transpupillary thermotherapy (TTT) has been thought to selectively damage the CNV by hyperthermia, inducing thrombotic vessel occlusion while sparing the overlying retina [5].

Heat shock proteins function as intra-cellular chaperones for other proteins and play a critical role in protein-protein interactions, assist in generating proper protein conformation and prevent pathological protein aggregation. Hsp proteins are expressed under physiological condition in all organisms and play an essential role in protein maintenance [6,7].

Hsp70's are a family of proteins that, as other heat shock proteins, aid in protein folding and stabilization. Hsp70's have been extensively investigated and are overexpressed under conditions of stress and serve to protect proteins from damage during stress [8,9].

Hsp70 is present in the retina [10] and Desmettre et al. [11] has shown that application of subtreshold transpupillary TTT irradiation induces overexpression of Hsp70 in a rabbit model of CNV. Here we have investigated whether CNV membranes from AMD patients express Hsp70 and whether the expression is modulated by pre-treatment with TTT or PDT.

## Methods

**Figure 1:**
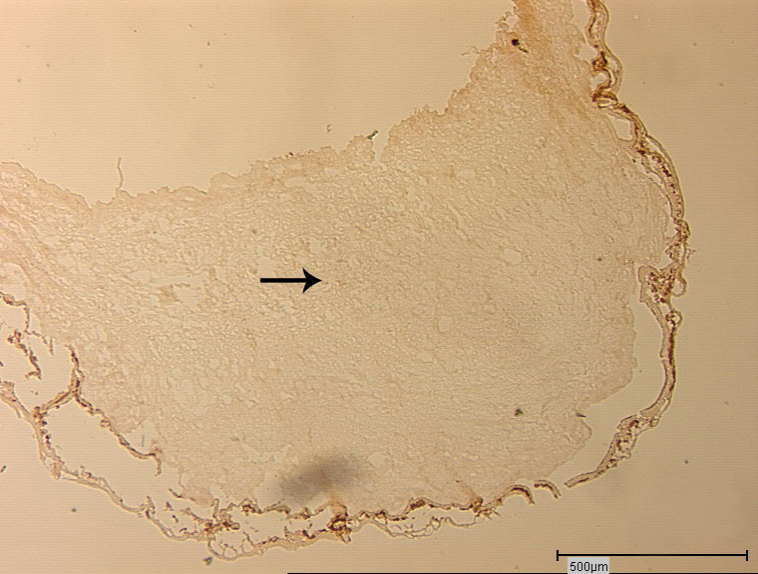
**Native cnv membrane**. Native cnv membrane (bright field, magnification 5×). Arrow indicates fibrovascular tissue.

Subretinal CNV tissue (Figure 1) was removed by pars plana vitrectomy and maintained at -24°C until use. The CNV tissue was embedded in Jung's medium and sectioned in 5 μm thick sections using a cryostat (Leitz Lauda Kryostat 1720 Digital). The sections were mounted on SuperFrost Plus glass slides, incubated with blocking solution (5% BSA in PBS) for 1 hour at room temperature in a humidified chamber followed by incubation with an anti-Hsp/Hsc70 monoclonal antibody (Chemicon^® ^International, Lot: 24060368). After overnight incubation at 4°C (or 1 hour at 37°C) the sections and HeLa cells (atcc number: CCL-2, LGC Promochem) were washed with PBS (Tween 20) 2 times for 5 minutes and incubated for 1 hour with a biotinylated anti-mouse secondary antibody (Polylink, Lot: C036, DCS Innovative Diagnostic Systems). After washing twice for 5 minutes with PBS (Tween 20) the sections and the HeLa cells were incubated with streptavidin-alkaline phosphatase for 30 min at room temperature followed by 2 × 5 minutes washes with PBS (tween 20), followed by incubation with the chromogenic alkaline phosphatase. An avidin-biotin blocking kit was used to inhibit endogenous avidin and biotin (BioGenex Laboratories). To inhibit endogenous alkaline phosphatase levamisol was used (Dako^®^).

**Figure 2:**
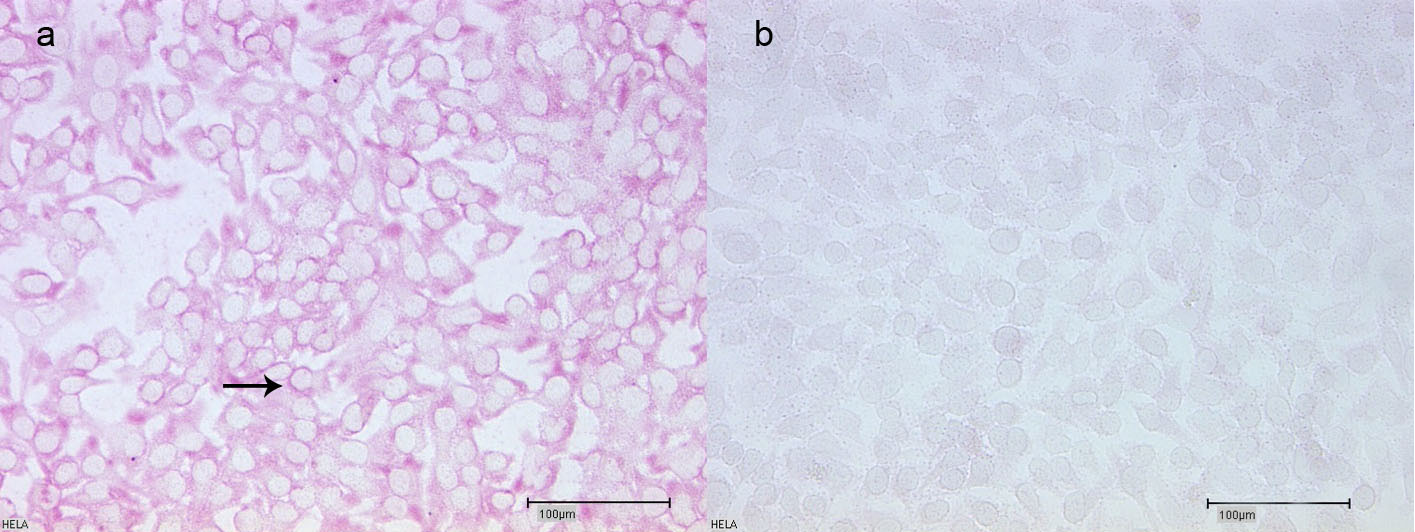
**HeLa Cells - HeLa Cells served as positive controls**. a) Nearly confluently growing cells. As primary antibody Anti Hsp 70 was used. Pink staining verifies presence of Hsp 70 (magnification 20×). Arrow: It appears that Hsp is localized mostly on the nuclear membrane. b) No primary antibody was used. Missing immunoreactivity verifies antibody specifity (magnification 20 x).

**Figure 3:**
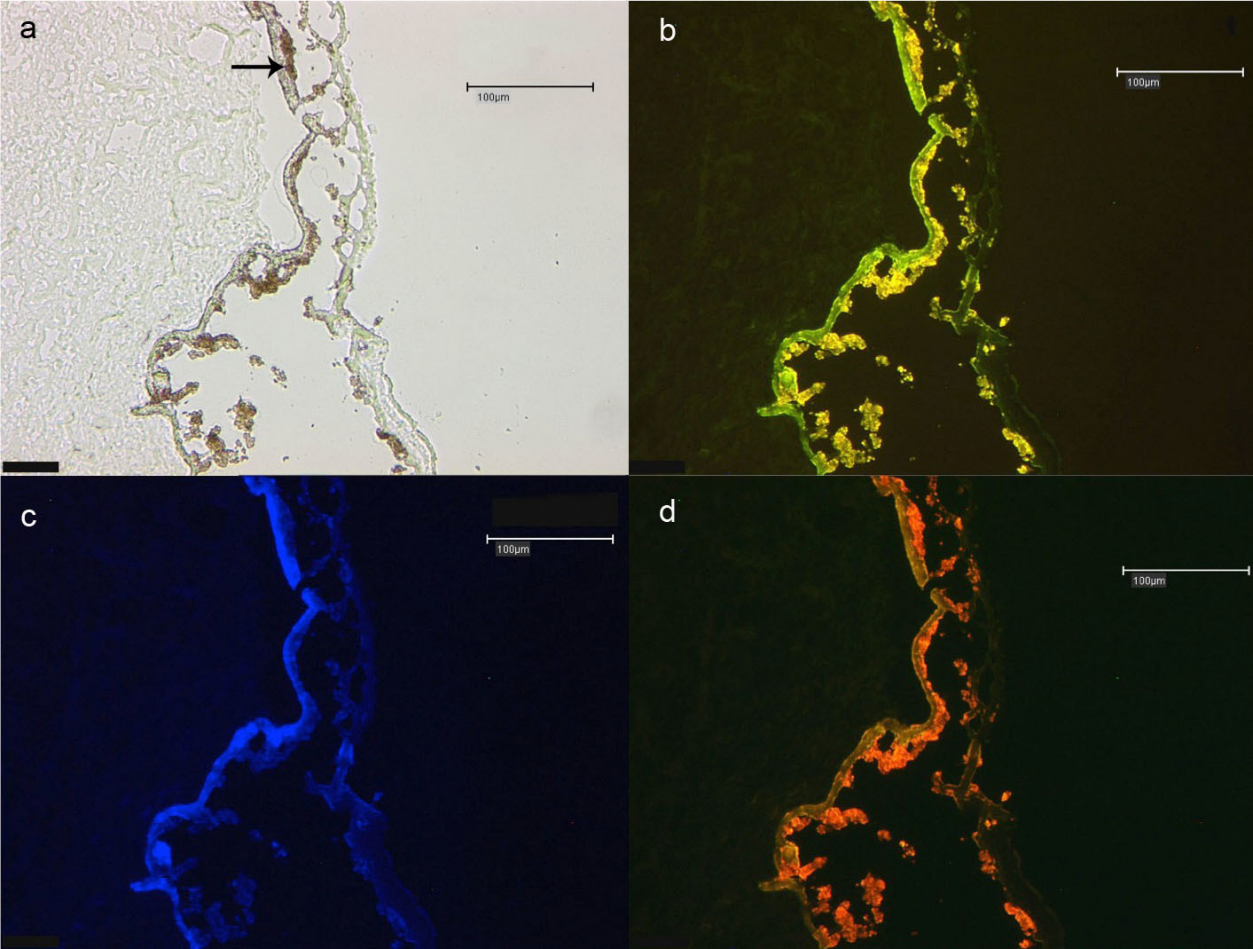
**Tissue autofluorescence**. a - d) These figures show four non treated (native) CNV membranes at20 × magnification. a) Shows bright field and Fig 3b-d the autofluorescence of lipofuscin in red, green and blue field. Although different wave length were used, tissue autofluorescence is present at various wavelengths so that nucleus staining (DAPI) and Hsp70-detection could not be performed. Arrow indicates the retinal pigment epithelium (RPE).

For negative controls (Figure 2), the primary antibody was replaced by PBS. CNV membranes were stained with Celestine and Eosin and embedded in Imagen embedding medium (Dako Cytomation). Due to broad spectrum tissue autofluorescene labeled antibodies could not be used (Figure 3).

For light microscopic evaluation a Leica DM IRB microscope was used. Images were captured with a microscope-mounted camera (Hitachi 3CCD HV-C20 AMP Color Camera) and processed with the PC software, DISKUS microscopic documentation (Hilgers, Germany).

## Results

**Figure 4:**
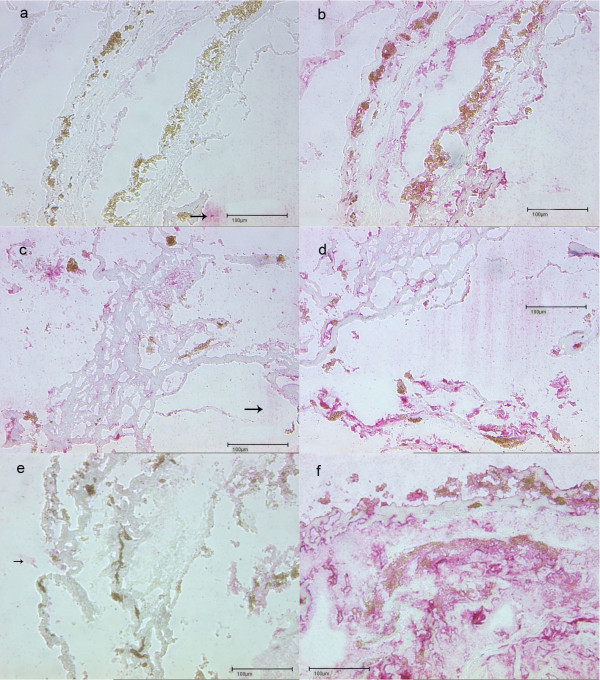
**CNV membranes with PDT-/TTT- and non- pretreatment**. PDT pretreated. a) PDT pretreated membrane which mostly consists of a fibrovascular. tissue architecture. No immunoreactivity (negative control) without primary antibody (Anti Hsp70, magnification 20 x). The surrounding pink staining reaction is an artefact (arrow). b) PDT pretreated membrane with immunoreactivity to the anti-Hsp70 primary antibody (pink stained tissue). TTT pretreated. c) TTT pretreated membrane which mostly consists of a fibrovascular tissue architecture. No immunoreactivity (negative control) without primary antibody (Anti Hsp70, magnification 20×). The surrounding pink staining reaction is an artefact (arrow). d) TTT pretreated membrane with immunoreactivity to anti Hsp70 (pink stained tissue). No pretreatment. e) Non pretreated membrane which mostly consists of a fibrovascular tissue architecture. No immunoreactivity (negative control). Without primary antibody (Anti Hsp70, magnification 20 x). The surrounding pink staining reaction is an artefact (arrow). f) Immunoreactivity to anti-HSP70 antibody of an untreated CNV membrane (pink stained tissue).

Of the fourteen CNV membranes from fourteen patients (7 female 72,5 ± 11,5 years and 6 male 73,5 ± 10,5 years) with AMD examined for the presence of Hsp/Hsc70, eleven membranes were Hsp70 positive, whereas 3 were Hsp/Hsc70 negative. The four membranes obtained from PDT pre-treated patients were all positive for Hsp/Hsc70 (Figure 4a, b) whereas of the two membranes obtained from TTT pre-treated patients one was positive and one was negative (Figure 4c, d). Of the eight membranes obtained from non-treated patient six were positive for Hsp/Hsc70 and two were negative (Figure 4e, f).

In summary 79% of the fourteen membranes examined Hsp/Hsc70 positive.

## Discussion and conclusions

The presence of Hsc70/Hsp70 has been described in the normal retina. Dean et al. [5] have shown that Hsc70 immunoreactivity was present in all layers of the retina, except in the photoreceptor outer segments and the retinal pigment epithelium (RPE) whereas Hsp70 immunoreactivity was restricted to the inner segments of photoreceptors, the membrana limitans externa and the outer nuclear layer. Dean et al. have suggested that different eye structures are dependent on different and specific heat shock proteins [12]. In our laboratory we have found that Hsp70 is normally present in all retinal layers, except the retinal pigment epithelium.

In this study Hsp70 was detected in the majority of CNV membranes secondary to AMD. The presence of significant amounts of Hsp70 in the CNV membranes indicates that the cellular components of the membranes are cells (fiboblasts, endothelial cells, RPE cells, etc.) that have been subjected to a stressful environment and inflammatory responses induced by CNV membrane in its surrounding tissue. Furthermore, heat shock proteins have been detected in various retinal layers in animal models, indicating a physiologic role in the eye. In a preliminary experiment, we tested our antibody in a human donor eye and detected Hsp70 in all retinal layers except the RPE. This could indicate a wider role of heat shock proteins in retinal physiology, but could also be due to retinal changes in a dying human, especially to a breakdown of the blood-retina barrier. Lafaut et al. [13] showed that the majority of classic CNV membranes in AMD patients consisted of subretinal, fibrovascular tissue and independent of the underlying disease, CNV membranes exhibit similar histological feature and can be seen as the result of unspecific proliferation of fibrovascular tissue. CNV membranes in our study showed similar histological features described by Lafaut [13], including the presence of sporadic vessels, in which the endothelium was positive for Hsp70.

Desmettre et al. [11] demonstrated an upregulation of heat shock proteins in the chorioretinal layers 24 hours after TTT treatment in a rabbit model. Unfortunately we had available only two membranes from patients that had previously been treated with TTT treatment and in addition the membranes were surgically excised weeks following TTT and it is therefore not possible to make any assessment as to whether TTT treatment in humans upregulates heat shock proteins.

## Acknowledgments

Supported by a grant (TV-B109) from the Interdisciplinary Centre for Clinical Research "BIOMAT." within the faculty of Medicine at the RWTH Aachen University and a grant by the START program of the medical faculty of the RWTH Aachen University.

## Consent

The study was performed with informed consent and following all the guidelines for experimental investigations required by the Institutional Review Board or Ethics Committee.

## Competing interests

The authors declare that they have no competing interests.

## Authors' contributions

APWJ: carried out histochemistry, histology and prepared a draft of the manuscript. DC: carried out histology and standardized immunostaining tests. GT: harvested CNV membranes, drafted and edited the manuscript. PW: harvested CNV membranes, drafted MS. AWAW: designed and coordinated study. All authors read and approved the final manuscript.

## References

[B1] Bressler NM Bressler SB Fine SL Age-related macular degeneration Surv Ophthalmol 1988 6 375 413 10.1016/0039-6257(88)90052-5 2457955

[B2] Pauleikhoff D Holz FG Age-related macular degeneration. 1. Epidemiology, pathogenesis and differential diagnosis Ophthalmologe 1996 3 299 315 8753995

[B3] Rosenfeld PJ Brown DM Heier JS MARINA Study Group. Ranibizumab for neovascular age-related macular degeneration N Engl J Med 2006 355 1419 1431 10.1056/NEJMoa054481 17021318

[B4] Ghazi NG Kirk TQ Knape RM Tiedeman JS Conway BP Is monthly retreatment with intravitreal bevacizumab (Avastin) necessary in neovascular age-related macular degeneration? Clin Ophthalmol 2010 4 307 14 10.2147/OPTH.S8598 20463798 PMC2861937

[B5] Dean DO Kent CR Tytell M Constitutive and inducible heat shock protein 70 immunoreactivity in the normal rat eye Invest Ophthalmol Vis Sci 1999 12 2952 62 10549657

[B6] Walter S Buchner J Molecular chaperones--cellular machines for protein folding Angewandte Chemie (International ed. in English) 2002 41 1098 113 10.1002/1521-3773(20020402)41:7<1098::AID-ANIE1098>3.0.CO;2-9 12491239

[B7] Borges JC Ramos CH Protein folding assisted by chaperones Protein and peptide letters 2005 12 257 61 10.2174/0929866053587165 15777275

[B8] Wegele H Müller L Buchner J Hsp70 and Hsp90 - a relay team for protein folding Rev. Physiol. Biochem. Pharmacol 2004 151 1 44 full_text 14740253 10.1007/s10254-003-0021-1

[B9] Beere HM Wolf BB Cain K Mosser DD Mahboubi A Kuwana T Tailor P Morimoto RI Cohen GM Green DR eat-shock protein 70 inhibits apoptosis by preventing recruitment of procaspase-9 to the Apaf-1 apoptosome" Nat. Cell Biol 2000 2 469 75 10.1038/35019501 10934466

[B10] Mainster MA Reichel E Transpupillary thermotherapy for age-related macular degeneration: long-pulse photocoagulation, apoptosis, and heat shock proteins Ophthalmic Surg Lasers 2000 5 359 73 11011704

[B11] Desmettre T Maurage CA Mordon S Heat shock protein hyperexpression on chorioretinal layers after transpupillary thermotherapy Invest Ophthalmol Vis Sci 2001 12 2976 80 11687545

[B12] Dean DO Tytell M Hsp25 and -90 immunoreactivity in the normal rat eye Invest Ophthalmol Vis Sci 2001 12 3031 40 11687552

[B13] Lafaut BA Bartz-Schmidt KU Vanden Broecke C Aisenbrey S De Laey JJ Heimann K Clinicopathological correlation in exudative age related macular degeneration: histological differentiation between classic and occult choroidal neovascularisation Br J Ophthalmol 2000 3 239 43 10.1136/bjo.84.3.239 PMC1723392 10684831

